# The Validity of Daily Self-Assessed Perceived Stress Measured Using Smartphones in Healthy Individuals: Cohort Study

**DOI:** 10.2196/13418

**Published:** 2019-08-19

**Authors:** Helga Þórarinsdóttir, Maria Faurholt-Jepsen, Henrik Ullum, Mads Frost, Jakob E Bardram, Lars Vedel Kessing

**Affiliations:** 1 The Copenhagen Affective Disorder Research Centre Psychiatric Center Copenhagen Copenhagen Denmark; 2 Department of Clinical Immunology Copenhagen University Hospital Copenhagen Denmark; 3 Monsenso ApS Valby Denmark; 4 Copenhagen Center for Health Technology Technical University of Denmark Lyngby Denmark

**Keywords:** emotional stress, smartphone, ecological momentary assessment, mobile phone, self-report, healthy individuals

## Abstract

**Background:**

Smartphones may offer a new and easy tool to assess stress, but the validity has never been investigated.

**Objective:**

This study aimed to investigate (1) the validity of smartphone-based self-assessed stress compared with Cohen Perceived Stress Scale (PSS) and (2) whether smartphone-based self-assessed stress correlates with neuroticism (Eysenck Personality Questionnaire-Neuroticism, EPQ-N), psychosocial functioning (Functioning Assessment Short Test, FAST), and prior stressful life events (Kendler Questionnaire for Stressful Life Events, SLE).

**Methods:**

A cohort of 40 healthy blood donors with no history of personal or first-generation family history of psychiatric illness and who used an Android smartphone were instructed to self-assess their stress level daily (on a scale from 0 to 2; beta values reflect this scale) for 4 months. At baseline, participants were assessed with the FAST rater-blinded and filled out the EPQ, the PSS, and the SLE. The PSS assessment was repeated after 4 months.

**Results:**

In linear mixed-effect regression and linear regression models, there were statistically significant positive correlations between self-assessed stress and the PSS (beta=.0167; 95% CI 0.0070-0.0026; *P*=.001), the EPQ-N (beta=.0174; 95% CI 0.0023-0.0325; *P*=.02), and the FAST (beta=.0329; 95% CI 0.0036-0.0622; *P*=.03). No correlation was found between smartphone-based self-assessed stress and the SLE.

**Conclusions:**

Daily smartphone-based self-assessed stress seems to be a valid measure of perceived stress. Our study contains a modest sample of 40 healthy participants and adds knowledge to a new but growing field of research. Smartphone-based self-assessed stress is a promising tool for measuring stress in real time in future studies of stress and stress-related behavior.

## Introduction

### Background

Stress is a common experience that occurs when an individual perceives that the environmental demands exceed his or her adaptive capacity [1]. At a European level, stress has been defined as “a state, which is accompanied by physical, psychological or social complaints or dysfunctions and which results from individuals feeling unable to bridge a gap with the requirement or expectations placed on them [2].” Stress can be categorized as *distress*, which is the unpleasant type of stress, or *eustress*, which is the good kind of stress, the type that motivates one to deal with whatever is causing the stress [3]. Stress can also be categorized as acute or chronic. Acute stress is short-lived, often relating to a specific stimulus or an event. It is accompanied by physical symptoms such as quickening heartbeat, muscular tensions, shortness of breath, and sweating. Chronic stress, on the other hand, is a long-term reaction to the pressures of daily life [4]. Over time, people may get used to the physical symptoms of chronic stress, but overexposure of the body to stress hormones can have long-term health effects. Stress affects the body in various ways: it can suppress the immune system, impact memory, and disturb digestion [5]. Chronic stress has been associated with cardiovascular disease [6], breast cancer [7], and psychiatric disorders [8]. High stress levels have also been found to be associated with higher all-cause mortality in men [9].

Stress is an individual experience, and even though the physical symptoms of stress (such as increased heart rate) can be measured objectively [10], most measures of stress focus on the individual’s perception of stress, that is, subjective stress. Individuals appraise situations and responses to stress differently. Measures of self-assessed stress vary from simple yes or no questions (“do you feel stressed?”) to a more complex grading of stress, for example, different point Likert-scales, to specific questions about stressful events (for a review, see [11]).

A more definite measure of subjective stress can be determined by using special instruments, such as stress assessment scales. Stress assessment scales consist of a list of questions relating to stressful events and experiences. By having individuals respond to specific questions, it is more likely that they are assessing the same kind of stress, that is, the kind of stress that has been defined by the assessment scale.

Cohen Perceived Stress Scale (PSS) is widely used for measuring individual perception of stress. It is commonly implemented in a 10-question form and measures the way respondents have found their lives unpredictable, uncontrollable, and overwhelming in the previous 14 days. The PSS has a good internal reliability (Cronbach alpha=.78-.91) and is correlated with various self-report and behavioral criteria, such as stressful life events and depressive symptoms. [12,13].

Some predictors of subjective stress have been identified in individual studies. Thus, female gender, low self-esteem, and neuroticism have consistently been associated with higher levels of subjective stress [14-17]. Having experienced stressful life events is also a predictor of higher levels of subjective stress [18].

In the last decade, with the release of smartphones and tablets, the use of mobile health (mHealth) has been steadily growing. mHealth is the practice of using mobile devices in medicine and public health [19]. One method of collecting health measures, such as subjective stress, on a mobile device is ecological momentary assessment. Ecological momentary assessment is a collection of methods used to collect “assessments of the subjects’ current or recent states, sampled repeatedly over time, in their natural environment” [20]. Smartphones are a convenient and nonintrusive tool to measure subjective stress, as most people carry their phones with them throughout the day and are used to interacting with it in many locations, in many situations, and at all times [21]. Subjective stress measured on smartphones could, therefore, potentially reflect a person’s real-time stress level. Being able to self-assess one’s own stress levels in real time can help individuals to be aware their own stress levels. Awareness of one’s own stress level is the first step toward coping with it [22]. Nevertheless, as previously reported in a systematic review by the authors, the validity of smartphone-based self-assessment of stress has never been systematically investigated [11].

### Objectives

The objective of this study was to investigate the validity of smartphone-based self-assessed stress evaluated daily. More specifically, the aims were to evaluate (1) the validity of smartphone-based self-assessed stress compared with the PSS and (2) whether smartphone-based self-assessed stress correlates with neuroticism and psychosocial functioning and whether prior stressful life events predict smartphone-based self-assessed stress.

## Methods

### Design and Settings

This study was conducted at The Copenhagen Affective Disorder Research Centre, Psychiatric Centre Copenhagen, Denmark, using a prospective design. To increase participation, the study only consisted of 1 physical visit at baseline, whereas follow-up was conducted via mail. Participants were recruited by approaching blood donors in the waiting room at the Blood Bank at Rigshospitalet, Copenhagen, at random occasions from November 2015 to August 2016. Inclusion criteria were as follows: individuals older than 18 years, no history of psychiatric illnesses, no first-generation history of psychiatric illnesses, and should have an Android smartphone as their regular smartphone. Exclusion criteria were pregnancy and lack of Danish language skills.

A study protocol was written in August 2015 and can be acquired by contacting the author. No changes were made to the study design during follow-up.

This cohort of healthy individuals was recruited as a control group for ongoing case-control studies, investigating differences between patients with bipolar disorder, healthy individuals at risk of bipolar disorder, and healthy individuals [23].

The Monsenso app used to evaluate stress was installed on the participants’ Android smartphones at baseline, and all participants were encouraged to carry their smartphones with them during the day and to use their smartphone as usual during the 4-month study period.

### Measures

**Figure 1:**
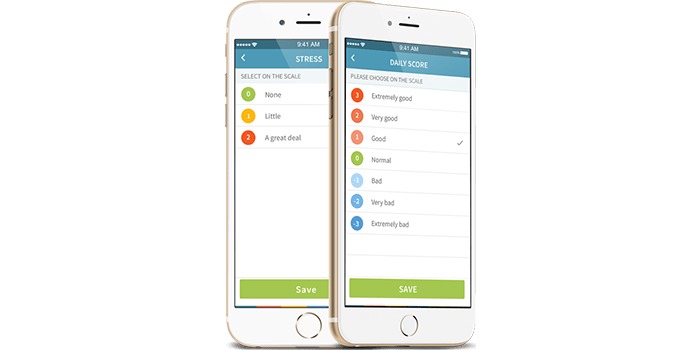
Examples of the self-assessment screenshot from the Monsenso app.

The Monsenso app is a smartphone app previously developed and investigated in a number of studies by the authors (eg, [23-25]). The app allowed participants to enter self-assessment data and includes automatically collected sensor data, such as measures of smartphone use, physical activity, and voice features. Participants were asked to self-assess parameters that are of importance in bipolar illness, such as mood, sleep, cognitive impairment, and stress. Both types of data could be historically visualized on the screen, allowing the participants to see their own data. Self-assessments were daily, and the app came with a preset alarm at 8 pm to remind participants to enter their data (Figure 1). Furthermore, analyses of the automatically collected sensor data and other self-assessment measures will be described in future papers.

### Baseline Assessments

#### Clinical Assessments

The participants were included in the study from February to August 2016 and participated for a 4-month long study period. Absence of any psychiatric diagnoses according to the International Classification of Diseases, Tenth Revision was confirmed using *Schedules for Clinical assessment in Neuropsychiatry* (SCAN) interviews [26]. All participants were assessed at baseline using the *Hamilton Depression Rating Scale-17 item* (HDRS-17) [27], the *Young Mania Rating Scale* (YMRS) [28], and the *Functioning Assessment Short Test* (FAST) [29]. Sociodemographic data on the participants were also collected at baseline.

#### Questionnaires

Participants filled out the following questionnaires at baseline: the *Eysenck Personality Questionnaire* (EPQ) [30], the *Kendler Questionnaire for Stressful Life Events* (SLE) [31], and the PSS [13].

### Follow-Up Assessments

At the end of the 4-month study period, participants received the PSS questionnaire [13] by mail, filled it out, and sent it back to the researchers. Participants could then uninstall the Monsenso app from their smartphones.

### Smartphone-Based Self-Assessed Stress

The Monsenso app prompted participants daily to self-evaluate stress. Stress was evaluated on a 3-point Likert scale with the 3 possible answers being 0=no stress, 1=little stress, and 2=much stress (Figure 1). Participants were encouraged to self-evaluate stress at the end of their day during the follow-up period.

### Questionnaire-Based Measures of Stress

Participants filled out the PSS at both baseline and follow-up. As the study period was longer than 14 days (as captured by the PSS), the questionnaire was repeated to account for variation over time and to increase the statistical power of the study. The PSS is a self-evaluating questionnaire comprising 10 items on the appraisal of situations as stressful in the last 14 days.

The questionnaire asks the participants to evaluate how often they have felt their lives to be unpredictable, uncontrollable, or overwhelming in the last 14 days. Participants responded on a 5-point scale ranging from 0 (never) to 4 (very often). A total of 4 items were worded in a positive direction and were therefore reverse scored. Total scores are from a scale of 0 to 40. A survey of healthy individuals in 2009 reported a mean (SD) PSS score of 15.52 (7.44) for men and 16.14 (7.56) for women [12].

### Additional Measures

Participants also filled out the EPQ and the SLE questionnaires at baseline. As the EPQ and the SLE regard long-time measures of personality and life events, respectively, they were not repeated at follow-up.

The EPQ-Neuroticism (EPQ-N) refers to the neuroticism score in the EPQ. The EPQ is a questionnaire with 100 yes or no questions. Different questions make up the total scores for the different personality traits: neuroticism, extroversion, and psychoticism. The EPQ has been shown to be strongly replicable across 34 countries [32], and the neuroticism score has good internal reliability (mean 0.83) [33]. Individuals who score high on neuroticism are more likely to be emotionally unstable than the average person and experience feelings such as anxiety, worry, guilt, and loneliness [34].

The SLE is a questionnaire about stressful life events [35,36]. It is divided into 2 parts: the first part is about stressful life events throughout an individual’s lifetime, and the second part is about stressful life events in the past 12 months. A total of 2 total scores are calculated from the 2 parts. Inter-rater reliability values have been shown to be good to excellent ranging from 0.82 to 0.93 [35,37].

At baseline, the FAST was used to assess functional impairment. The FAST is an interviewer-administrated instrument that comprises 24 questions divided into 6 specific areas of functioning **[29].** These are autonomy, occupational functioning, cognitive functioning, financial issues, interpersonal relationships, and leisure time. Total scores are on a scale from 0 to 72, and higher scores indicate more functional impairments. Evaluations using the FAST scale were conducted rater-blinded by an interviewer without access to smartphone data.

### Statistical Methods

First, descriptive analyses were produced (percentages, means, and SDs), and afterward the a priori defined statistical analyses were computed using linear regression models and linear mixed-effect regression models. A 2-level linear mixed-effect regression model, which accommodates both variation of the variables of interest within participants (intraindividual variation) and between participants (interindividual variation), was employed. The model included a fixed effect of visit number (baseline and follow-up) and a participant-specific random effect, allowing for individual intercept and slope for each participant. For all analyses, we first considered an unadjusted analysis and second, an analysis adjusted for sex and age as predefined possible confounding covariables.

Data were assembled into 3 different datasets, as the various measures and questionnaires addressed different time periods. In the first dataset, an average of self-assessed stress over the first and last 14 days of the study, was used as the PSS addresses the previous 14 days. In the second dataset, an average of all self-assessed stress data over the 4-month study period was used as the SLE, and the EPQ-N do not address a specific time period. Finally, in the third dataset, an average of self-assessed stress over the first 7 days of the study was used as the FAST at baseline addresses the previous 7 days. Even though questionnaires at baseline refer to the previous 7 or 14 days, we have decided to use the first 7 or 14 days of self-assessed measures, as we did not have any measures before baseline.

The data collected were entered in Microsoft Excel sheets, and the statistical software program Stata version 13.1 (StataCorp) was used for the statistical analyses. The statistical significance limit was set at *P*<.05.

### Ethical Approval

The study was approved by the regional ethics committee in the capital region of Denmark (H-7-2014-007) and the Danish Data Protection Agency. All potential participants received written and oral information before informed consent was obtained, and participants were informed that they could withdraw from the study at any time during the study. The study complied with the Helsinki declaration [38].

## Results

### Participants’ Flow, Background Characteristics, and Questionnaires

**Figure 2:**
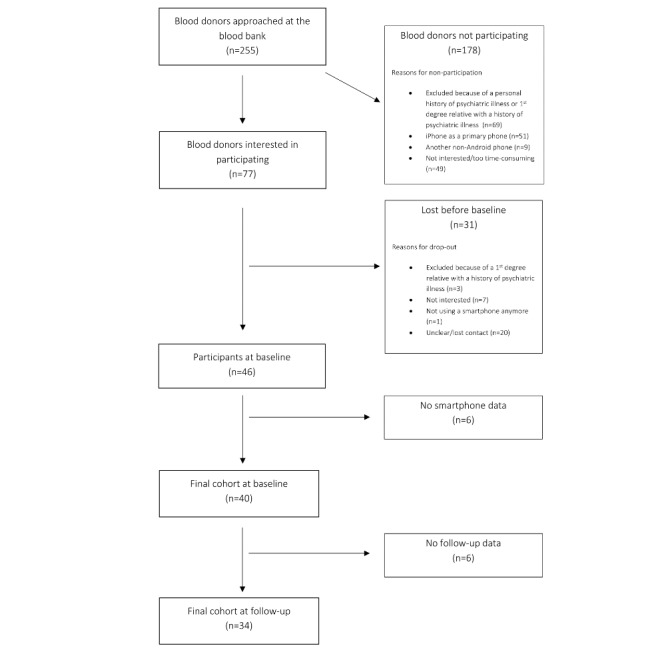
Flow diagram of participants recruited from Rigshospitalets blood bank from November 2015 to August 2016.

A total of 255 individuals were approached at the Blood Bank during the 9-month recruitment period. Over half of those (129) were ineligible to participate, and another 49 were not interested in participating in the study or thought it would be too time consuming. Of the remaining 77 individuals, 46 were included in the study. Of these, 6 were not included in the final cohort because of a lack of smartphone data. Additional 6 participants did not return the follow-up questionnaire (see flow diagram, Figure 2).

Thus, the final cohort in this study consisted of 40 healthy blood donors. The mean age was 35.24 (SD 12.79) years; 55% (22/40) of them were women, and 65% (26/40) of them were in a relationship. Information on background and sociodemographic characteristics of participants is shown in Table 1.

The mean score of self-assessed stress on smartphones was 0.12 (SD 0.34) measured on a scale from 0 to 2. Scores from questionnaires at baseline and follow-up are shown in Table 2. Participants had an average of 81.82 (SD 38.83) self-assessment days. There was no difference in the PSS and smartphone-based self-assessed stress between sexes (*P*>.82). There was no association between the age of the participants and the PSS (*P*>.54), but there was a statistically significant positive association (beta=.002; 95% CI 0.001-0.003; *P*<.001) between smartphone-based self-assessed stress and the age of the participants, namely, that for every 10-year increase in age, there was an increase in smartphone-based self-assessed stress by 0.02 on a scale from 0 to 2.

### The Validity of Smartphone-Based Self-Assessed Stress Compared With Perceived Stress Scale

Table 3 presents the results of linear mixed-effect regression model for the self-assessed stress using smartphones and the sum scores on the PSS.

As can be seen, a statistically significant positive correlation was found between smartphone-based self-assessed stress and the PSS in both the unadjusted model and the model adjusted for age and sex (unadjusted model beta=.0167; 95% CI 0.0070-0.0026; *P*=.001), indicating that for every 10-point increase on the PSS, the smartphone-based self-assessed stress increased 0.167 on a scale from 0 to 2. Overall, there was little to no difference between the unadjusted and the adjusted models.

### Association Between Smartphone-Based Self-Assessed Stress and Neuroticism

Table 3 presents the results of linear mixed-effect regression model for the self-assessed stress using smartphones and the sum scores on the EPQ-N.

A statistically significant positive correlation was found between smartphone-based self-assessed stress and the neuroticism score on the EPQ in both the unadjusted model and the model adjusted for age and sex (unadjusted model beta=.0174; 95% CI 0.0023-0.0325; *P*=.02), indicating that for every score that increased 10 points on the EPQ-N, the smartphone-based self-assessed stress was 0.174 higher on a scale from 0 to 2.

### Association Between Smartphone-Based Self-Assessed Stress and Functioning Assessment Short Test

Table 3 presents the results of linear mixed-effect regression model for the self-assessed stress using smartphones and the sum scores on the FAST.

A statistically significant positive correlation was found between smartphone-based self-assessed stress and the FAST in both the unadjusted model and the model adjusted for age and sex (unadjusted model beta=.0329; 95% CI 0.0036-0.0622; *P*=.03), indicating that for every score that increased 10 points on the FAST, the smartphone-based self-assessed stress was 0.329 higher on a scale from 0 to 2.

### Association Between Smartphone-Based Self-Assessed Stress and Prior Stressful Life Events

Analysis of the correlation between smartphone-based self-assessed stress and prior SLE, measured with Kendler SLE Questionnaire, yielded no statistically significant results. Neither the SLE in the previous year before baseline (unadjusted model beta=–.0055; 95% CI –0.0329 to 0.0219; *P*=.69) nor the SLE over lifetime (unadjusted model beta=.0064; 95% CI –0.0373 to 0.0502; *P*=.77) correlated with smartphone-based self-assessed stress.

**Table 1:** Background and sociodemographic characteristics of study participants (N=40).

Characteristics		Statistics	
Age (years), mean (SD)		35.24 (12.79)	
Female gender, n (%)		22 (55)	
**Occupation, n (%)**			
	Employed	23 (57.5)
	Unemployed	2 (5)
	Student	15 (37.5)
Education (years), mean (SD)		14.77 (2.09)	
Sick days, median (IQR^a^)		2 (1-4.5)	
**Civil status, n (%)**			
	Alone	22 (55)
	Cohabiting	18 (45)
**Marital status, n (%)**			
	Never married	32 (80)
	Married	7 (17.5)
	Divorced	1 (2.5)
**Civil partner, n (%)**			
	In a relationship	26 (65)
	Single	14 (35)
**Smoking, n (%)**			
	Smoker	8 (20)
	Former smoker	6 (15)
	Never smoked	26 (65)
**Alcohol units per week, median (IQR)**			
	Total	5 (2-7.5)
	Female gender	4 (1-6)
	Male gender	6 (3-12)
Former alcohol abuse, n (%)		1 (2.5)	
**Use of cannabis, n (%)**			
	Never	36 (90)
	<1 monthly	4 (10)
Height (centimeters), median (IQR)		174. 68 (9.69)	
Weight (kilograms), median (IQR)		78.22 (13.91)	

^a^IQR: interquartile range.

**Table 2:** Total scores on different questionnaires and smartphone-based self-assessed stress.

Questionnaires and measures (score range; N)		Scores	
EPQ-N^a^ (0-14; N=38), median (IQR^b^)		4.5 (2-7)	
**PSS^c^**			
	Baseline (0-20; N=37), median (IQR)	6 (4-9)
	Follow-up (0-21; N=34), median (IQR)	5.5 (3-9)
SLE^d^, last 12 months (0-10; N=38), median (IQR)		2 (1-4)	
SLE, lifetime (0-5; N=38), median (IQR)		1 (0-2)	
FAST^e^ (0-8; N=40), median (IQR)		1 (0-3)	
Smartphone-based self-assessed stress (0-2; N=40), mean (SD)		0.12 (0.34)	

^a^EPQ-N: Eysenck Personality Questionnaire—Neuroticism.

^b^IQR: interquartile range.

^c^PSS: Perceived Stress Scale.

^d^SLE: Kendler Questionnaire for Stressful Life Events.

^e^FAST: Functioning Assessment Short Test.

**Table 3:** Correlations between smartphone-based self-assessed stress and the Perceived Stress Scale, the Eysenck Personality Questionnaire-Neuroticism, the Functioning Assessment Short Test, and the Kendler Stressful Life Events questionnaire (N=39).

Stress (scale 0-2)	Unadjusted		Adjusted^a^	
	Coefficient (95% CI)	*P* value	Coefficient (95% CI)	*P* value
PSS^b^ (n=39)	0.0167 (0.0070 to 0.0026)	.001	0.0162 (0.0064 to 0.0259)	.001
EPQ-N^c^ (n=38)	0.0174 (0.0023 to 0.0325)	.02	0.0167 (0.0011 to 0.0323)	.04
FAST^d^ (n=38)	0.0329 (0.0036 to 0.0622)	.03	0.0307 (0.0012 to 0.6018)	.04
SLE^e^, last year (n=36)	–0.0055 (–0.0329 to 0.0219)	.69	–0.0191 (–0.0520 to 0.0138)	.25
SLE, lifetime (n=36)	0.0064 (–0.0373 to 0.0502)	.77	–0.0020 (–0.0483 to 0.0444)	.93

^a^Adjusted for age and sex.

^b^PSS: Perceived Stress Scale.

^c^EPQ-N: Eysenck Personality Questionnaire-Neuroticism.

^d^FAST: Functioning Assessment Short Test.

^e^SLE: Kendler Questionnaire for Stressful Life Events

### Additional Analyses

Finally, additional analyses of the correlation between the PSS and the EPQ-N, the FAST, and the SLE, respectively, were made to assess the internal validity between the PSS and smartphone-based self-assessed stress. A statistically significant positive correlation was found between the PSS and the EPQ-N (unadjusted model beta=.8663*,* 95% CI 0.6362-1.0965*, P*=.001; adjusted model beta=.8770*,* 95% CI 0.6413-1.1128*, P*=.001), indicating that for every score that increased 1 on the EPQ-N, the PSS score was 0.87 higher on a scale from 0 to 40. A statistically significant positive correlation was also found between the PSS and the FAST (unadjusted model beta=1.0965, 95% CI 0.4229-1.7702, *P*=.001; adjusted model beta=1.095, 95% CI 0.4208-1.7699, *P*=.001), indicating that for every score that increased 1 on the FAST, the PSS score was 1.09 higher on a scale from 0 to 40.

As found in the models using smartphone-based self-assessed stress, no statistically significant correlations were found between the PSS and the SLE. Neither the SLE in the previous year (unadjusted model *P*=.06; adjusted model *P*=.08), nor the SLE over lifetime (unadjusted model *P*=.95; adjusted model *P*=.77), correlated with the PSS scores.

## Discussion

### Principal Findings

This study followed 40 healthy blood donors for 4 months with daily self-assessment of stress using their smartphone. We found statistically significant positive correlations between smartphone-based self-assessed stress, and the PSS, the EPQ-N, and the FAST, respectively. Smartphone-based self-assessed stress did not correlate with prior stressful life events, neither in the previous year nor over a lifetime. Thus, smartphone-based self-assessed stress was validly evaluated as compared with the PSS. Furthermore, increased smartphone-based self-assessed stress was associated with increased neuroticism and decreased functioning.

To the best of our knowledge, this is the first study to explicitly investigate the validity of smartphone-based self-assessed stress. The findings from this study indicate that smartphone-based self-assessed stress is a valid measure of subjective stress on its own. As previously reported in a systematic review by the authors on smartphone-based self-assessment of stress in healthy adult individuals, 2 other previous studies have investigated the correlation between smartphone-based self-assessed stress and the PSS [11]. A study by Wang et al [39] on college students reported a statistically significant positive correlation between smartphone-based self-assessed stress and the PSS [39]. However, the objective of the study was to investigate associations to automatic objective sensor data from smartphones. Thus, Wang et al [39] did not investigate stress as the primary objective of the study, and all data were collected either on the smartphone or online, with no interviewer-administrated measures, thus increasing the risk of chance findings. Adams et al reported a nonsignificant correlation between the 2 measures of stress in a small sample (n=7) of graduate students and postdoctoral researchers [40].

The finding, that increased smartphone-based self-assessed stress was associated with increased neuroticism, adds to the validity of self-assessed stress using smartphones. Neuroticism is a personality trait and can be defined as a temperamental trait of emotionality; a tendency to arouse quickly when stimulated and to inhibit slowly when aroused [41,42]. Neuroticism is generally associated with a higher level of subjective stress and a tendency to inefficiently cope with stress [14,43-45]. People with high neuroticism scores are generally more at risk for developing psychiatric disorders, such as mood and anxiety disorders, sometimes called neurotic or stress-related disorders [46]. Awareness of one’s stress level could potentially be important in people who have high neuroticism scores as it could help them to cope with stress better.

Furthermore, the finding, that increased smartphone-based self-assessed stress was associated with decreased functioning, also adds to the validity of self-assessed stress using smartphones. We used the FAST in our study, a rater-blinded measure for psychosocial functioning, and this study is the first one in this field of research to have used such a measure. Psychosocial function is an important measure, as an individual’s function is essential for being able to take care of oneself and one’s family. Very high levels of stress, such as those seen in posttraumatic stress disorder, lead to psychosocial functional impairment, including social and occupational impairment [47]. The relationship between stress and psychosocial function has primarily been investigated in patient populations, but studies including healthy controls find the same relationship, namely that higher stress levels are associated with impairments of psychosocial functioning, such as social functioning [48]. Stress levels have also been found to predict level of disability later in life [49].

In this study, we did not find a significant correlation between smartphone-based self-assessed stress and prior stressful life events. The SLE does not distinguish between dependent and independent stressful life events, and thus, the total score comprised all kinds of stressful life events. Independent stressful life events are those that are not influenced by the individual (eg, death of a relative), whereas dependent stressful life events are those that are in some way influenced by the individual (eg, a fight with a loved one) [50]. Dependent stressful life events are the ones that are associated with stress and depressive symptoms [51]. It is also possible that our participants, who have low average scores on the PSS, the EPQ-N, and the FAST, are better at coping with stressful life events than the general population.

Additional analyses of our data showed that higher scores on the PSS were associated with higher scores on the EPQ-N and the FAST, but no relationship was found between the PSS and the SLE. The same pattern was found in the primary analyses of the study: higher scores on smartphone-based self-assessed stress were associated with higher scores on the EPQ-N and the FAST, but no relationship was found between smartphone-based self-assessed stress and the SLE. This suggests that even though our 2 measures of stress are different in form, they are in fact measuring the same phenomenon, subjective stress.

### Advantages

This study was the first study to investigate the validity of smartphone-based self-assessed stress. It was also 1 of the first studies in a relatively new field of research that did not focus primarily on the technical side of the smartphone system. Another strength of the study is that it uses ecological momentary assessment to collect measures of self-assessed stress on a daily basis and therefore minimizes recall bias [20]. A further strength of the study is that participating individuals were not aware of the aims and focus on stress in this report, hereby decreasing the risk of false-positive associations between self-reported measures (smartphone-based self-assessed stress vs the PSS and the EPQ-N, respectively), as individuals were recruited as control individuals for reported and ongoing studies [23]. The Monsenso app has been used in previous studies and has shown to have high usability [52,53]. Participants received no economic compensation for participating in the study and used their own smartphones and were, therefore, already familiar with the devices and were used to interacting with them. We used different interviewer-administrated and validated measures at baseline to ensure that our participants were healthy (no SCAN diagnosis of mental illness, no depressive or manic symptoms according to the HDRS-17 and YMRS) and had a rater-blinded measure of psychosocial function (FAST). Both unadjusted analyses and analyses adjusted for sex and age were presented.

### Limitations

There are also some limitations to this study that should be mentioned. Our participants were recruited from the Blood Bank at Rigshospitalet and are likely to represent a *super healthy* population group [54]. Similarly, bias may have been introduced both in the selection of active participation and in the loss of follow-up or incomplete data. Baseline data on the PSS did not differ between participants lost to follow-up and participants with complete data (*P*>.72). Participants had an average of 81.82 (SD 38.83) days with self-assessment, which amounts to 68.2% (81.82/120) adherence. An adherence of close to 100% would be optimal but is difficult to achieve. By using smartphones, we were able to get an accurate measure of adherence as participants were not able to self-assess retrospectively. Only individuals with an Android-based smartphone as their main smartphone were recruited to the study. This was because of technical reasons but could have introduced bias to the study. This affected the sample size of the study as well. A recent study investigated Android and iPhone users and found that they differed only a little in personality, but research in this area is scarce, and knowledge is limited [55]. It would be optimal to include participants using all kinds of smartphones in future studies.

### Perspectives and Implications

As smartphone ownership has grown over the past decade, digital phenotyping has become a new and promising research field. Smartphones generate a high amount of data that can be collected in real time [56] and, thus, have the potentiality to reflect an individual’s current state.

Stress is an increasing public health problem, and chronic exposure to stress is a risk factor for developing mental and physical diseases [6-8]. Awareness of one’s own stress level is important as it is the first step toward coping with it [22], and smartphones are an unobtrusive and easily accessible tool for this. Self-assessment of daily stress using smartphones may be a step toward stress awareness.

Having a valid measure of subjective stress on a smartphone makes it possible to investigate further stress and stress-related behavior. Understanding more details regarding stress is a possible step toward decreasing it, and as stress is an individual experience, it is important that individuals are aware of their own stress level and stressors. It is possible that self-monitoring of subjective stress may help decrease stress levels, but it has never been investigated. The alternative, that self-monitoring of subjective stress increases stress levels, is a possibility as well. This should be investigated in further studies.

It is important that future studies using smartphone-based self-assessed stress also use previously validated measures of stress, such as the PSS, to confirm the validity of their smartphone-based self-assessed stress.

### Conclusions

This study investigated the validity of smartphone-based self-assessed stress in relation to scores on the PSS, as well as the association between smartphone-based self-assessed stress and neuroticism, psychosocial functioning, and prior stressful life events, respectively. Smartphone-based self-assessed stress was a valid measure of subjective stress and correlated with the EPQ-N and the FAST. Measuring subjective stress on a smartphone represents a new and promising way to measure perceived stress in real time. As a valid measure of stress, smartphone-based self-assessed stress can be used in future studies of stress and stress-related behavior and may be a step toward stress awareness.

## Abbreviations

EPQ-N: Eysenck Personality Questionnaire-Neuroticism
FAST: Functioning Assessment Short Test
HDRS-17: Hamilton Depression Rating Scale-17 item
mHealth: mobile health
PSS: Perceived Stress Scale
SCAN: Clinical assessment in Neuropsychiatry
SLE: Kendler Questionnaire for Stressful Life Events
YMRS: Young Mania Rating Scale

## References

[1] Cohen S, Gordon LU, Kessler RC (1997). Measuring Stress: A Guide for Health and Social Scientists.

[2] (2010). European Foundation for the Improvement of Living and Working Conditions.

[3] Selye H (1976). The Stress of Life. Revised Edition.

[4] (2018). American Psychological Association.

[5] Yaribeygi H, Panahi Y, Sahraei H, Johnston TP, Sahebkar A (2017). The impact of stress on body function: a review. Excli J.

[6] Steptoe A, Kivimäki M (2012). Stress and cardiovascular disease. Nat Rev Cardiol.

[7] Kruk J (2012). Self-reported psychological stress and the risk of breast cancer: a case-control study. Stress.

[8] Plieger T, Melchers M, Montag C, Meermann R, Reuter M (2015). Life stress as potential risk factor for depression and burnout. Burn Res.

[9] Nielsen NR, Kristensen TS, Schnohr P, Grønbaek M (2008). Perceived stress and cause-specific mortality among men and women: results from a prospective cohort study. Am J Epidemiol.

[10] Föhr T, Tolvanen A, Myllymäki T, Järvelä-Reijonen E, Rantala S, Korpela R, Peuhkuri K, Kolehmainen M, Puttonen S, Lappalainen R, Rusko H, Kujala UM (2015). Subjective stress, objective heart rate variability-based stress, and recovery on workdays among overweight and psychologically distressed individuals: a cross-sectional study. J Occup Med Toxicol.

[11] Þórarinsdóttir H, Kessing LV, Faurholt-Jepsen M (2017). Smartphone-based self-assessment of stress in healthy adult individuals: a systematic review. J Med Internet Res.

[12] Cohen S, Janicki-Deverts D (2012). Who's stressed? Distributions of psychological stress in the United States in probability samples from 1983, 2006, and 2009. J Appl Soc Psychol.

[13] Cohen S, Kamarck T, Mermelstein R (1983). A global measure of perceived stress. J Health Soc Behav.

[14] Gramstad TO, Gjestad R, Haver B (2013). Personality traits predict job stress, depression and anxiety among junior physicians. BMC Med Educ.

[15] Juth V, Smyth JM, Santuzzi AM (2008). How do you feel? Self-esteem predicts affect, stress, social interaction, and symptom severity during daily life in patients with chronic illness. J Health Psychol.

[16] Saleh D, Camart N, Romo L (2017). Predictors of stress in college students. Front Psychol.

[17] Wolf L, Stidham AW, Ross R (2015). Predictors of stress and coping strategies of US accelerated vs generic Baccalaureate nursing students: an embedded mixed methods study. Nurse Educ Today.

[18] Feizi A, Aliyari R, Roohafza H (2012). Association of perceived stress with stressful life events, lifestyle and sociodemographic factors: a large-scale community-based study using logistic quantile regression. Comput Math Methods Med.

[19] World Health Organization (2011). mHealth: New Horizons for Health Through Mobile Technologies.

[20] Shiffman S, Stone AA, Hufford MR (2008). Ecological momentary assessment. Annu Rev Clin Psychol.

[21] Raento M, Oulasvirta A, Eagle N (2009). Smartphones: an emerging tool for social scientists. Sociol Methods Res.

[22] Shapiro SL, Schwartz GE, Bonner G (1998). Effects of mindfulness-based stress reduction on medical and premedical students. J Behav Med.

[23] Faurholt-Jepsen M, Busk J, Þórarinsdóttir H, Frost M, Bardram JE, Vinberg M, Kessing LV (2019). Objective smartphone data as a potential diagnostic marker of bipolar disorder. Aust N Z J Psychiatry.

[24] Bardram JE, Frost M, Szántó K, Faurholt-Jepsen M, Vinberg M, Kessing LV (2013). Designing Mobile Health Technology for Bipolar Disorder: A Field Trial of the Monarca System. Proceedings of the SIGCHI Conference on Human Factors in Computing Systems.

[25] Faurholt-Jepsen M, Frost M, Ritz C, Christensen EM, Jacoby AS, Mikkelsen RL, Knorr U, Bardram JE, Vinberg M, Kessing LV (2015). Daily electronic self-monitoring in bipolar disorder using smartphones - the MONARCA I trial: a randomized, placebo-controlled, single-blind, parallel group trial. Psychol Med.

[26] Wing JK, Babor T, Brugha T, Burke J, Cooper JE, Giel R, Jablenski A, Regier D, Sartorius N (1990). SCAN. Schedules for clinical assessment in neuropsychiatry. Arch Gen Psychiatry.

[27] Hamilton M (1967). Development of a rating scale for primary depressive illness. Br J Soc Clin Psychol.

[28] Young R, Biggs J, Ziegler V, Meyer D (1978). A rating scale for mania: reliability, validity and sensitivity. Br J Psychiatry.

[29] Rosa AR, Sánchez-Moreno J, Martínez-Aran A, Salamero M, Torrent C, Reinares M, Comes M, Colom F, van Riel W, Ayuso-Mateos JL, Kapczinski F, Vieta E (2007). Validity and reliability of the functioning assessment short test (FAST) in bipolar disorder. Clin Pract Epidemiol Ment Health.

[30] Eysenck HJ (1975). Manual of the Eysenck Personality Questionnaire (Junior and Adult).

[31] Kendler KS, Karkowski LM, Prescott CA (1998). Stressful life events and major depression: risk period, long-term contextual threat, and diagnostic specificity. J Nerv Ment Dis.

[32] Barrett PT, Petrides KV, Eysenck SB, Eysenck HJ (1998). The Eysenck Personality Questionnaire: an examination of the factorial similarity of P, E, N, and L across 34 countries. Pers Individ Dif.

[33] Caruso JC, Witkiewitz K, Belcourt-Dittloff A, Gottlieb JD (2016). Reliability of scores from the Eysenck Personality Questionnaire: a reliability generalization study. Educ Psychol Meas.

[34] Tackett JL, Lahey BB, Widiger TA (2017). Neuroticism. The Oxford Handbook of the Five Factor Model.

[35] Kendler KS, Kessler RC, Walters EE, MacLean C, Neale MC, Heath AC, Eaves LJ (1995). Stressful life events, genetic liability, and onset of an episode of major depression in women. Am J Psychiatry.

[36] Vinberg M, Mortensen E, Kyvik K, Kessing L (2007). Personality traits in unaffected twins discordant for affective disorder. Acta Psychiatr Scand.

[37] Kendler KS, Gardner CO (2010). Dependent stressful life events and prior depressive episodes in the prediction of major depression: the problem of causal inference in psychiatric epidemiology. Arch Gen Psychiatry.

[38] World Medical Association (2013). World Medical Association declaration of Helsinki: ethical principles for medical research involving human subjects. J Am Med Assoc.

[39] Wang R, Chen F, Chen Z, Li T, Harari G, Tignor S, Zhou X, Ben-Zeev D, Campbell AT (2014). StudentLife: Assessing Mental Health, Academic Performance and Behavioral Trends of College Students Using Smartphones. Proceedings of the 2014 ACM International Joint Conference on Pervasive and Ubiquitous Computing.

[40] Adams P, Rabbi M, Rahman T, Matthews M, Voida A, Gay G, Choudhury T, Voida S (2014). Towards Personal Stress Informatics: Comparing Minimally Invasive Techniques for Measuring Daily Stress in the Wild. Proceedings of the 8th International Conference on Pervasive Computing Technologies for Healthcare.

[41] Eysenck M (1985). Personality and Individual Differences: A Natural Science Approach.

[42] McCrae RR, John OP (1992). An introduction to the five-factor model and its applications. J Pers.

[43] Abbasi IS (2016). The role of neuroticism in the maintenance of chronic baseline stress perception and negative affect. Span J Psychol.

[44] Dawson BF, Thompson NJ (2017). The effect of personality on occupational stress in veterinary surgeons. J Vet Med Educ.

[45] Schneider TR (2004). The role of neuroticism on psychological and physiological stress responses. J Exp Soc Psychol.

[46] Kotov R, Gamez W, Schmidt F, Watson D (2010). Linking 'big' personality traits to anxiety, depressive, and substance use disorders: a meta-analysis. Psychol Bull.

[47] Rodriguez P, Holowka DW, Marx BP (2012). Assessment of posttraumatic stress disorder-related functional impairment: a review. J Rehabil Res Dev.

[48] Grove TB, Tso IF, Chun J, Mueller SA, Taylor SF, Ellingrod VL, McInnis MG, Deldin PJ (2016). Negative affect predicts social functioning across schizophrenia and bipolar disorder: findings from an integrated data analysis. Psychiatry Res.

[49] Kulmala J, von Bonsdorff MB, Stenholm S, Törmäkangas T, von Bonsdorff ME, Nygård CH, Klockars M, Seitsamo J, Ilmarinen J, Rantanen T (2013). Perceived stress symptoms in midlife predict disability in old age: a 28-year prospective cohort study. J Gerontol A Biol Sci Med Sci.

[50] Kendler KS, Karkowski LM, Prescott CA (1999). The assessment of dependence in the study of stressful life events: validation using a twin design. Psychol Med.

[51] Harkness KL, Monroe SM, Simons AD, Thase M (1999). The generation of life events in recurrent and non-recurrent depression. Psychol Med.

[52] Faurholt-Jepsen M, Vinberg M, Frost M, Christensen EM, Bardram JE, Kessing LV (2015). Smartphone data as an electronic biomarker of illness activity in bipolar disorder. Bipolar Disord.

[53] Frost M, Doryab A, Faurholt-Jepsen M, Kessing LV, Bardram JE (2013). Supporting Disease Insight Through Data Analysis: Refinements of the Monarca Self-Assessment System. Proceedings of the 2013 ACM International Joint Conference on Pervasive and Ubiquitous Computing.

[54] Schwartz S, Susser E (2011). The use of well controls: an unhealthy practice in psychiatric research. Psychol Med.

[55] Götz FM, Stieger S, Reips UD (2017). Users of the main smartphone operating systems (iOS, Android) differ only little in personality. PLoS One.

[56] Torous J, Staples P, Onnela JP (2015). Realizing the potential of mobile mental health: new methods for new data in psychiatry. Curr Psychiatry Rep.

